# 1-[(Butyl­amino)(phen­yl)meth­yl]naphthalen-2-ol

**DOI:** 10.1107/S1600536811000067

**Published:** 2011-01-12

**Authors:** Hen-Mei Ni

**Affiliations:** a College of Chemistry and Chemical Engineering, Southeast University, Nanjing 211189, People’s Republic of China

## Abstract

In the title compound, C_21_H_23_NO, obtained *via* a one-pot synthesis, an intra­molecular O—H⋯N hydrogen bond stabilizes the mol­ecular conformation. The dihedral angle between the fused ring system and the phenyl ring is 78.27 (5)°. The crystal packing is characterized by helical chains of mol­ecules linked by C—H⋯O hydrogen bonds.

## Related literature

For applications of Betti-type reactions, see: Zhao *et al.* (2004 ▶); Lu *et al.* (2002 ▶); Xu *et al.* (2004 ▶); Wang *et al.* (2005 ▶)
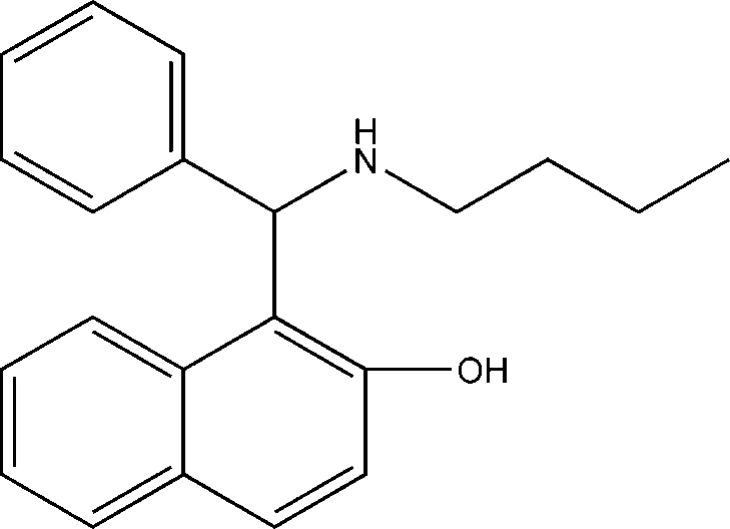


## Experimental

### 

#### Crystal data

C_21_H_23_NO
*M* *_r_* = 305.40
Orthorhombic, 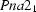

*a* = 10.842 (7) Å
*b* = 16.651 (7) Å
*c* = 9.787 (6) Å
*V* = 1766.9 (17) Å^3^
*Z* = 4
Mo *K*α radiation
μ = 0.07 mm^−1^
*T* = 293 K
0.30 × 0.25 × 0.15 mm

#### Data collection

Rigaku Mercury2 diffractometer
Absorption correction: multi-scan (*CrystalClear*; Rigaku, 2005 ▶) *T*_min_ = 0.856, *T*_max_ = 1.000
14129 measured reflections
3121 independent reflections
1998 reflections with *I* > 2σ(*I*)
*R*_int_ = 0.078

#### Refinement

*R*[*F* ^2^ > 2σ(*F* ^2^)] = 0.087
*wR*(*F* ^2^) = 0.134
*S* = 1.02
3121 reflections
211 parameters
2 restraints
H-atom parameters constrained
Δρ_max_ = 0.25 e Å^−3^
Δρ_min_ = −0.13 e Å^−3^

### 

Data collection: *CrystalClear* (Rigaku, 2005 ▶); cell refinement: *CrystalClear*; data reduction: *CrystalClear*; program(s) used to solve structure: *SHELXS97* (Sheldrick, 2008 ▶); program(s) used to refine structure: *SHELXL97* (Sheldrick, 2008 ▶); molecular graphics: *SHELXTL* (Sheldrick, 2008 ▶); software used to prepare material for publication: *SHELXTL*.

## Supplementary Material

Crystal structure: contains datablocks I, global. DOI: 10.1107/S1600536811000067/gw2088sup1.cif

Structure factors: contains datablocks I. DOI: 10.1107/S1600536811000067/gw2088Isup2.hkl

Additional supplementary materials:  crystallographic information; 3D view; checkCIF report

## Figures and Tables

**Table 1:** Hydrogen-bond geometry (Å, °)

*D*—H⋯*A*	*D*—H	H⋯*A*	*D*⋯*A*	*D*—H⋯*A*
O1—H1*A*⋯N1	0.82	1.89	2.580 (5)	142

**Fig. 1.**
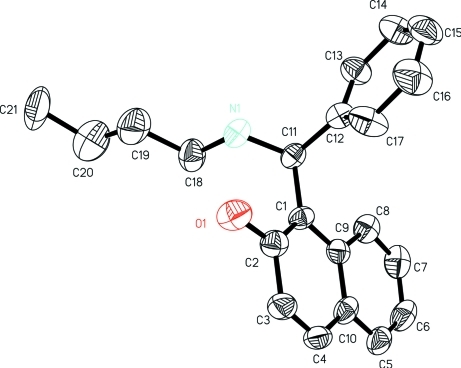
Perspective structure of the title compound, showing the atom-numbering scheme. Displacement ellipsoids are drawn at the 30% probability level.

**Fig. 2.**
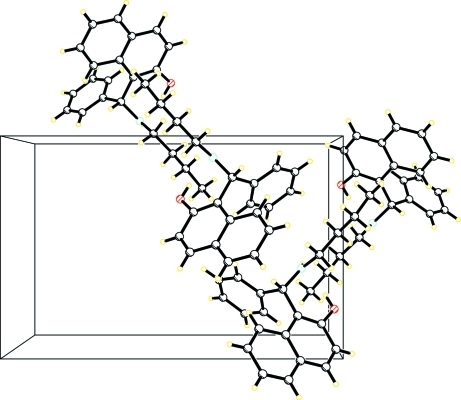
The crystal packing of the title compound viewed along the c axis showing the two-dimensionnal hydrogen bondings network.

## supplementary crystallographic information

### Comment

Over one hundred years ago, Betti developed a straightforward synthesis involving the condensation of 2-naphthol, ammonia and equivalents of benzaldehyde, followed by the addition of HCl and KOH to yield 1-(a-aminobenzyl)-2-naphthol. This product which possesses an asymmetric carbon center is known as a Betti base (Zhao & Li *et al.* 2004). Betti-type reaction is an important method to synthesize chiral ligands and by this method many unnatural homochiral amino-phenol compounds have been obtained (Lu *et al.* 2002; Xu *et al.* 2004; Wang *et al.* 2005). Here we report the synthesis and crystal structure of the title compound (Fig. 1), obtained by a three-component condensation reaction of 2-naphthol, benzaldehyde and butan-1-amine under solvent-free condition.

Molecules of the title compound have normal geometric parameters. The bond lengths and angles are within their normal ranges. The rings A (C1–C10) and B (C12–C17) are, of course, planar and the dihedral angle between them is A/B = 78.27 (5). As can be seen from the packing diagram (Fig. 2), the intramolecular O—H···N hydrogen bond seems to be effective in the stabilization of the crystal structure. Dipole–dipole and van der Waals interactions are effective in the molecular packing.

### Experimental

benzaldehyde (1.59 g, 0.015 mol) and butan-1-amine (1.095 g, 0.015 mol) was added to 2-naphthol (2.16 g, 0.015 mol) without solvent under nitrogen. The temperature was raised to 120°C in one hour gradually and the mixture was stirred at this temperature for 10 h. The system was treated with 20 ml of ethanol 95% and cooled. The precipitate was filtered and washed with a small amount of ethanol 95%. The title compound was isolated using column chromatography (Petroleum ether: ethyl acetate-2:1). Single crystals suitable for X-ray diffraction analysis were obtained from slow evaporation of ethyl acetate solution.

### Refinement

H atoms bonded to O atoms were located in a difference map and refined with distance restraints of O—H = 0.82 Å, and with *U*_iso_(H) = 1.368Ueq(O). Other H atoms were positioned geometrically and refined using a riding model, with C—H = 0.93–0.98 Å and *U*_iso_(H) = 1.3–1.6*U*_eq_(C). The structure does not contain a strong anomalous scatterer, therefore MERG 3 have been applied. 1459 Friedel pairs were merged.

### Crystal data

**Table:**

C_21_H_23_NO	*F*(000) = 656
*M**_r_* = 305.40	*D*_x_ = 1.148 Mg m^−^^3^
Orthorhombic, *P**n**a*2_1_	Mo *K*α radiation, λ = 0.71073 Å
Hall symbol: P 2c -2n	Cell parameters from 2229 reflections
*a* = 10.842 (7) Å	θ = 2.4–27.4°
*b* = 16.651 (7) Å	µ = 0.07 mm^−^^1^
*c* = 9.787 (6) Å	*T* = 293 K
*V* = 1766.9 (17) Å^3^	Prism, colorless
*Z* = 4	0.30 × 0.25 × 0.15 mm

### Data collection

**Table:**

Rigaku Mercury2 diffractometer	3121 independent reflections
Radiation source: fine-focus sealed tube	1998 reflections with *I* > 2σ(*I*)
graphite	*R*_int_ = 0.078
Detector resolution: 13.6612 pixels mm^-1^	θ_max_ = 25.0°, θ_min_ = 2.4°
CCD_Profile_fitting scans	*h* = −12→12
Absorption correction: multi-scan (*CrystalClear*; Rigaku, 2005)	*k* = −19→19
*T*_min_ = 0.856, *T*_max_ = 1.000	*l* = −11→11
14129 measured reflections	

### Refinement

**Table:**

Refinement on *F*^2^	Secondary atom site location: difference Fourier map
Least-squares matrix: full	Hydrogen site location: inferred from neighbouring sites
*R*[*F*^2^ > 2σ(*F*^2^)] = 0.087	H-atom parameters constrained
*wR*(*F*^2^) = 0.134	*w* = 1/[σ^2^(*F*_o_^2^) + (0.0007*P*)^2^ + 1.9999*P*] where *P* = (*F*_o_^2^ + 2*F*_c_^2^)/3
*S* = 1.02	(Δ/σ)_max_ < 0.001
3121 reflections	Δρ_max_ = 0.25 e Å^−^^3^
211 parameters	Δρ_min_ = −0.13 e Å^−^^3^
2 restraints	Extinction correction: *SHELXL97* (Sheldrick, 2008), Fc^*^=kFc[1+0.001xFc^2^λ^3^/sin(2θ)]^-1/4^
Primary atom site location: structure-invariant direct methods	Extinction coefficient: 0.0043 (9)

### Special details

**Table:**

Geometry. All e.s.d.'s (except the e.s.d. in the dihedral angle between two l.s. planes) are estimated using the full covariance matrix. The cell e.s.d.'s are taken into account individually in the estimation of e.s.d.'s in distances, angles and torsion angles; correlations between e.s.d.'s in cell parameters are only used when they are defined by crystal symmetry. An approximate (isotropic) treatment of cell e.s.d.'s is used for estimating e.s.d.'s involving l.s. planes.
Refinement. Refinement of *F*^2^ against ALL reflections. The weighted *R*-factor *wR* and goodness of fit *S* are based on *F*^2^, conventional *R*-factors *R* are based on *F*, with *F* set to zero for negative *F*^2^. The threshold expression of *F*^2^ > σ(*F*^2^) is used only for calculating *R*-factors(gt) *etc*. and is not relevant to the choice of reflections for refinement. *R*-factors based on *F*^2^ are statistically about twice as large as those based on *F*, and *R*- factors based on ALL data will be even larger.

### Fractional atomic coordinates and isotropic or equivalent isotropic displacement parameters (Å^2^)

**Table:**

	*x*	*y*	*z*	*U*_iso_*/*U*_eq_	
O1	0.2175 (3)	0.0230 (2)	0.4209 (4)	0.0825 (11)	
H1A	0.2786	0.0483	0.3966	0.124*	
N1	0.4017 (4)	0.1176 (2)	0.4649 (5)	0.0718 (12)	
H1D	0.4572	0.1535	0.4201	0.086*	
C1	0.1998 (4)	0.1346 (3)	0.5773 (5)	0.0551 (12)	
C2	0.1594 (4)	0.0636 (3)	0.5250 (5)	0.0657 (14)	
C3	0.0511 (5)	0.0240 (3)	0.5748 (5)	0.0717 (16)	
H3A	0.0260	−0.0245	0.5368	0.086*	
C4	−0.0136 (5)	0.0574 (4)	0.6767 (6)	0.0748 (16)	
H4A	−0.0832	0.0311	0.7097	0.090*	
C5	−0.0463 (5)	0.1666 (4)	0.8420 (6)	0.0822 (18)	
H5A	−0.1145	0.1397	0.8769	0.099*	
C6	−0.0147 (6)	0.2386 (5)	0.8958 (6)	0.093 (2)	
H6A	−0.0610	0.2609	0.9662	0.111*	
C7	0.0874 (6)	0.2788 (4)	0.8448 (6)	0.0899 (19)	
H7A	0.1090	0.3282	0.8822	0.108*	
C8	0.1585 (5)	0.2473 (4)	0.7391 (5)	0.0739 (16)	
H8A	0.2257	0.2758	0.7055	0.089*	
C9	0.1277 (4)	0.1714 (3)	0.6832 (5)	0.0601 (13)	
C10	0.0212 (4)	0.1313 (3)	0.7350 (5)	0.0646 (14)	
C11	0.3145 (4)	0.1767 (3)	0.5238 (5)	0.0566 (12)	
H11A	0.3552	0.2032	0.6009	0.068*	
C12	0.2873 (4)	0.2400 (3)	0.4156 (5)	0.0603 (12)	
C13	0.1947 (6)	0.2301 (4)	0.3193 (6)	0.094 (2)	
H13A	0.1443	0.1849	0.3231	0.113*	
C14	0.1761 (6)	0.2872 (5)	0.2161 (7)	0.113 (2)	
H14A	0.1128	0.2807	0.1530	0.136*	
C15	0.2523 (6)	0.3530 (4)	0.2093 (7)	0.098 (2)	
H15A	0.2425	0.3901	0.1390	0.118*	
C16	0.3415 (6)	0.3644 (4)	0.3043 (6)	0.097 (2)	
H16A	0.3908	0.4100	0.3015	0.116*	
C17	0.3588 (5)	0.3071 (3)	0.4058 (6)	0.0789 (16)	
H17A	0.4215	0.3148	0.4694	0.095*	
C18	0.4708 (5)	0.0719 (4)	0.5698 (7)	0.0921 (19)	
H18A	0.4145	0.0401	0.6248	0.111*	
H18B	0.5153	0.1083	0.6294	0.111*	
C19	0.5652 (6)	0.0146 (5)	0.4899 (8)	0.127 (3)	
H19A	0.5187	−0.0218	0.4321	0.152*	
H19B	0.6169	0.0472	0.4312	0.152*	
C20	0.6390 (7)	−0.0296 (5)	0.5756 (10)	0.162 (4)	
H20A	0.5881	−0.0654	0.6297	0.194*	
H20B	0.6816	0.0064	0.6377	0.194*	
C21	0.7336 (6)	−0.0788 (5)	0.4962 (9)	0.153 (4)	
H21A	0.7833	−0.1090	0.5589	0.230*	
H21B	0.7854	−0.0434	0.4441	0.230*	
H21C	0.6916	−0.1149	0.4355	0.230*	

### Atomic displacement parameters (Å^2^)

**Table:**

	*U*^11^	*U*^22^	*U*^33^	*U*^12^	*U*^13^	*U*^23^
O1	0.079 (2)	0.084 (2)	0.085 (3)	−0.017 (2)	0.006 (2)	−0.019 (2)
N1	0.053 (2)	0.076 (3)	0.086 (3)	−0.001 (2)	0.009 (2)	−0.003 (3)
C1	0.045 (3)	0.070 (3)	0.050 (3)	−0.004 (2)	−0.003 (2)	0.003 (3)
C2	0.057 (3)	0.075 (4)	0.065 (4)	−0.001 (3)	−0.002 (3)	−0.001 (3)
C3	0.060 (3)	0.075 (4)	0.080 (4)	−0.010 (3)	−0.002 (3)	0.009 (3)
C4	0.053 (3)	0.091 (4)	0.080 (4)	−0.007 (3)	0.000 (3)	0.028 (3)
C5	0.065 (4)	0.110 (5)	0.072 (4)	0.021 (4)	−0.002 (3)	0.022 (4)
C6	0.075 (4)	0.128 (6)	0.075 (5)	0.031 (4)	0.010 (4)	−0.002 (4)
C7	0.100 (5)	0.095 (5)	0.074 (4)	0.024 (4)	−0.016 (4)	−0.013 (4)
C8	0.073 (4)	0.090 (4)	0.058 (4)	0.008 (3)	−0.014 (3)	−0.002 (3)
C9	0.050 (3)	0.074 (4)	0.057 (3)	0.001 (3)	−0.014 (2)	0.013 (3)
C10	0.053 (3)	0.087 (4)	0.054 (3)	0.016 (3)	−0.004 (3)	0.006 (3)
C11	0.047 (3)	0.064 (3)	0.059 (3)	−0.003 (2)	−0.004 (2)	−0.010 (3)
C12	0.059 (3)	0.067 (3)	0.055 (3)	−0.008 (3)	−0.002 (3)	−0.013 (3)
C13	0.108 (5)	0.103 (5)	0.072 (4)	−0.042 (4)	−0.023 (4)	0.017 (4)
C14	0.109 (5)	0.146 (7)	0.085 (5)	−0.022 (5)	−0.038 (4)	0.034 (5)
C15	0.125 (6)	0.104 (5)	0.066 (4)	−0.002 (5)	0.003 (4)	0.028 (4)
C16	0.119 (6)	0.084 (5)	0.088 (5)	−0.025 (4)	0.000 (4)	0.008 (4)
C17	0.080 (4)	0.075 (4)	0.082 (4)	−0.021 (3)	−0.012 (3)	0.010 (4)
C18	0.076 (4)	0.086 (4)	0.115 (5)	0.009 (3)	−0.012 (4)	−0.005 (4)
C19	0.077 (5)	0.151 (7)	0.152 (8)	0.026 (4)	−0.008 (5)	0.026 (6)
C20	0.136 (8)	0.188 (10)	0.161 (9)	0.033 (7)	−0.001 (7)	−0.012 (8)
C21	0.105 (6)	0.109 (6)	0.245 (11)	0.032 (5)	0.032 (6)	−0.047 (6)

### Geometric parameters (Å, °)

**Table:**

O1—C2	1.375 (6)	C11—H11A	0.9800
O1—H1A	0.8200	C12—C17	1.363 (6)
N1—C18	1.481 (6)	C12—C13	1.387 (6)
N1—C11	1.483 (5)	C13—C14	1.402 (8)
N1—H1D	0.9548	C13—H13A	0.9300
C1—C2	1.362 (6)	C14—C15	1.374 (8)
C1—C9	1.435 (7)	C14—H14A	0.9300
C1—C11	1.520 (6)	C15—C16	1.355 (8)
C2—C3	1.431 (6)	C15—H15A	0.9300
C3—C4	1.340 (7)	C16—C17	1.390 (8)
C3—H3A	0.9300	C16—H16A	0.9300
C4—C10	1.409 (7)	C17—H17A	0.9300
C4—H4A	0.9300	C18—C19	1.603 (8)
C5—C6	1.353 (8)	C18—H18A	0.9700
C5—C10	1.406 (8)	C18—H18B	0.9700
C5—H5A	0.9300	C19—C20	1.373 (9)
C6—C7	1.386 (8)	C19—H19A	0.9700
C6—H6A	0.9300	C19—H19B	0.9700
C7—C8	1.393 (7)	C20—C21	1.526 (9)
C7—H7A	0.9300	C20—H20A	0.9700
C8—C9	1.417 (7)	C20—H20B	0.9700
C8—H8A	0.9300	C21—H21A	0.9600
C9—C10	1.427 (6)	C21—H21B	0.9600
C11—C12	1.523 (6)	C21—H21C	0.9600
			
C2—O1—H1A	109.5	C17—C12—C11	120.4 (5)
C18—N1—C11	113.2 (4)	C13—C12—C11	122.0 (5)
C18—N1—H1D	108.8	C12—C13—C14	120.8 (6)
C11—N1—H1D	99.4	C12—C13—H13A	119.6
C2—C1—C9	117.8 (5)	C14—C13—H13A	119.6
C2—C1—C11	122.3 (4)	C15—C14—C13	119.4 (6)
C9—C1—C11	119.8 (4)	C15—C14—H14A	120.3
C1—C2—O1	123.9 (5)	C13—C14—H14A	120.3
C1—C2—C3	122.4 (5)	C16—C15—C14	120.4 (6)
O1—C2—C3	113.7 (5)	C16—C15—H15A	119.8
C4—C3—C2	119.5 (5)	C14—C15—H15A	119.8
C4—C3—H3A	120.2	C15—C16—C17	119.4 (6)
C2—C3—H3A	120.2	C15—C16—H16A	120.3
C3—C4—C10	121.6 (5)	C17—C16—H16A	120.3
C3—C4—H4A	119.2	C12—C17—C16	122.3 (6)
C10—C4—H4A	119.2	C12—C17—H17A	118.8
C6—C5—C10	121.9 (6)	C16—C17—H17A	118.8
C6—C5—H5A	119.1	N1—C18—C19	106.9 (5)
C10—C5—H5A	119.1	N1—C18—H18A	110.3
C5—C6—C7	119.3 (6)	C19—C18—H18A	110.3
C5—C6—H6A	120.3	N1—C18—H18B	110.3
C7—C6—H6A	120.3	C19—C18—H18B	110.3
C6—C7—C8	121.8 (6)	H18A—C18—H18B	108.6
C6—C7—H7A	119.1	C20—C19—C18	113.2 (7)
C8—C7—H7A	119.1	C20—C19—H19A	108.9
C7—C8—C9	119.5 (6)	C18—C19—H19A	108.9
C7—C8—H8A	120.2	C20—C19—H19B	108.9
C9—C8—H8A	120.2	C18—C19—H19B	108.9
C8—C9—C10	118.0 (5)	H19A—C19—H19B	107.8
C8—C9—C1	122.1 (5)	C19—C20—C21	111.6 (8)
C10—C9—C1	119.9 (5)	C19—C20—H20A	109.3
C5—C10—C4	121.9 (6)	C21—C20—H20A	109.3
C5—C10—C9	119.4 (6)	C19—C20—H20B	109.3
C4—C10—C9	118.8 (5)	C21—C20—H20B	109.3
N1—C11—C1	110.4 (4)	H20A—C20—H20B	108.0
N1—C11—C12	108.2 (4)	C20—C21—H21A	109.5
C1—C11—C12	113.6 (4)	C20—C21—H21B	109.5
N1—C11—H11A	108.1	H21A—C21—H21B	109.5
C1—C11—H11A	108.1	C20—C21—H21C	109.5
C12—C11—H11A	108.1	H21A—C21—H21C	109.5
C17—C12—C13	117.5 (5)	H21B—C21—H21C	109.5

### Hydrogen-bond geometry (Å, °)

**Table:**

*D*—H···*A*	*D*—H	H···*A*	*D*···*A*	*D*—H···*A*
O1—H1A···N1	0.82	1.89	2.580 (5)	142
